# Architectural and biochemical advances in understanding microbial CO_2_ concentrating mechanisms

**DOI:** 10.1111/tpj.70913

**Published:** 2026-05-11

**Authors:** Cornelia E. M. Flothow, Andreas M. Küffner

**Affiliations:** ^1^ Max Planck Institute for Multidisciplinary Sciences Am Faßberg 11 Göttingen 37077 Lower Saxony Germany

**Keywords:** carbonate transporters, carboxysomes, CO_2_ concentrating mechanisms, CO_2_ fixation, microbial organelles, one‐carbon metabolism, photosynthesis, pyrenoids, Rubisco

## Abstract

CO_2_ concentrating mechanisms (CCMs) are ubiquitous in phototrophs. They are found in the micro as well as the macro world. Different domains of life have developed strikingly different approaches to concentrate inorganic carbon around Rubisco, the most prevalent enzyme on earth. While prokaryotes have developed carboxysomes, icosahedral shell protein cages, microbial eukaryotic phototrophs such as green algae, on the other hand, have developed pyrenoids, liquid–liquid phase separated organelles. In contrast to carboxysomes and pyrenoids, which represent biophysical CCMs, some vascular plants (C4 & CAM plants) have developed biochemical ways of concentrating CO_2_ around Rubisco. In this review, we discuss advances in structural, biochemical, and mechanistic understanding of microbial CCMs in the last 5–10 years.

## Significance Statement

Microbial CO_2_ concentrating mechanisms (CCMs) are ubiquitous and essential for efficient carbon fixation in a wide variety of phototrophs; however, many aspects of their architecture, biogenesis, and evolutionary diversity remain incompletely understood. This review article highlights recent advances in carboxysome and pyrenoid biology, the role of carbonate transporters, and the molecular diversity of CCMs, providing a comprehensive overview to inform future efforts for engineering carbon fixation in phototrophs.

## INTRODUCTION

CO_2_ concentrating mechanisms (CCMs) represent one of the most innovative evolutionary solutions enabling autotrophic organisms to thrive despite fluctuating and often limiting environmental CO_2_ levels. CCMs are composed of integrated biochemical and structural adaptations, which enhance CO_2_ supply to the central Calvin–Benson–Bassham (CBB) cycle carboxylase Rubisco. Hence, CCMs overcome Rubisco's relatively low selectivity and catalytic rate (de Pins et al., 2025), which is specifically relevant in environments where dissolved CO_2_ is scarce.

The earliest evidence for CCMs arose from physiological studies in the mid‐20th century that observed unexpectedly high rates of photosynthesis and low rates of photorespiration in land plants. The C4 photosynthetic pathway, first proposed by Kortschak, Hatch, and Slack in the 1960s, was a major breakthrough (Hatch & Slack, 1966; Iglesias et al., 1987; Kortschak et al., 1965). It explained how specialized leaf anatomy and biochemical partitioning allow many terrestrial plants to pre‐concentrate CO_2_ around Rubisco, thus greatly improving efficiency under warm, dry conditions. The concept of the ‘CO_2_ pump’ subsequently surfaced in studies of aquatic microorganisms, where biochemical shuttles and cellular structures were hypothesized to elevate CO_2_ levels at the carboxylation sites (Kaplan & Reinhold, 1999; Rae et al., 2013; Shively, Ball, Brown, & Saunders, 1973).

The finding of CCMs in prokaryotic cyanobacteria and eukaryotic algae marked another milestone for understanding the most prevalent one‐carbon metabolism. In cyanobacteria, membrane transporters for inorganic carbon (C_i_; bicarbonate and CO_2_) and highly ordered protein microcompartments termed carboxysomes were discovered (Gantt & Conti, 1969). These structures encapsulate Rubisco together with a carbonic anhydrase (CA) and act as biophysical CCMs, creating microenvironments with locally elevated CO_2_ concentrations (Price & Badger, 1989a; Shively, Ball, & Kline, 1973; So et al., 2004). Meanwhile, most algae (i.e., green algae, red algae, diatoms, some dinoflagellates, haptophytes, cryptophytes, chlorarachniophytes, euglenids as well as a few brown algae and glaucophytes) and most hornwort species were shown to organize Rubisco into a distinct organelle known as the pyrenoid (Barrett et al., 2021). These organelles are coupled to thylakoid tubules that deliver CO_2_ generated by nearby CAs and can additionally be surrounded by a starch sheath or other encompassing barriers (Freeman Rosenzweig et al., 2017; Lacoste‐Royal & Gibbs, 1987; Mackinder et al., 2016; Ohad et al., 1967; Sager & Palade, 1957).

Molecular genetics in the late 20th and early 21st century enabled the identification and functional dissection of the many genes involved in CCM regulation (Price et al., 2008). Key discoveries included the transport proteins BicA and SbtA in cyanobacteria and the scaffolding protein Essential Pyrenoid Component 1 (EPYC1) in *Chlamydomonas*, which links Rubisco molecules to form pyrenoid matrices (Mackinder et al., 2016; Shibata et al., 2001). Genomic studies provided vital insights into CCM diversity across microbial lineages, revealing that carboxysome and pyrenoid‐like structures evolved multiple times, presumably in response to falling atmospheric CO_2_ levels throughout Earth's history (Flamholz & Shih, 2020; Long et al., 2024; Melnicki et al., 2021; Moromizato et al., 2024; Rae et al., 2013; Sandrini et al., 2014; Sommer et al., 2017; Villarreal & Renner, 2012; Whitehead et al., 2014).

While these organelles have been known for decades, the last two have seen remarkable progress in understanding the biochemical and structural mechanisms underpinning CCMs. Cryo‐electron tomography (cryo‐ET), proteomics, and live cell imaging have mapped the 3D organization of carboxysomes and pyrenoids with high resolution, revealing how phase separation, specialized shell proteins, and dynamic protein–protein interactions coordinate Rubisco packaging and catalytic activity (Engel et al., 2015; Freeman Rosenzweig et al., 2017; Huang et al., 2020; Ni et al., 2022; Wunder et al., 2018; Zhan et al., 2018). These technological advances revealed that CCM microcompartments operate as liquid‐like droplets or crystalline matrices, and how CA strategically converts bicarbonate to CO_2_ in close proximity to the active site of Rubisco (Blikstad et al., 2023; Meyer & Griffiths, 2013; Zang et al., 2021).

Biochemical reconstitution and synthetic biology have further illuminated CCM function by enabling the assembly of carboxysome and pyrenoid proteins in heterologous model systems, such as *Escherichia coli* or plant chloroplasts, respectively (Atkinson et al., 2016; Flamholz et al., 2020). By transplanting the CCM, it was possible to demonstrate its mechanistic sufficiency for conferring growth under ambient air and the required components for functionality, including specialized linkers and shell proteins, have been elucidated. Parallel work in plant biology explored the introduction of cyanobacterial and proteobacterial CCMs into C3 crops, with the aim of enhancing photosynthetic rates in order to increase crop yields in global agriculture (Chen et al., 2023; Hanson et al., 2016).

The *de novo* assembly, transplantation, and redesign of CCM components continue to be a primary focus for current research (Chen et al., 2023; Sun, Chen, et al., 2025; Trettel et al., 2025). Recent works have not only engineered carboxysomes in plant chloroplasts, but also manipulated the Rubisco‐binding modes of scaffold proteins, and optimized shell permeability for selective metabolite flux. Sophisticated genetic toolkits now allow for rapid construction, testing, and evolutionary improvement of CCM modules (Hennacy & Jonikas, 2020; Trettel et al., 2025).

In summary, understanding and engineering CCMs spans a broad spectrum of fields such as plant physiology, evolutionary biology, molecular genetics, and high‐resolution structural biochemistry. While early physiological puzzles revealed distinct solutions (i.e., carboxysomes versus pyrenoids) to Rubisco's shortcomings, each of them represents a blueprint for next‐generation carbon‐fixation engineering. Even though carboxysomes and pyrenoids have been discovered almost 75 years ago, CCM research remains a frontier, at the intersection of biology and biotechnology, for understanding how a putatively inefficient CO_2_ assimilating cycle (CBB cycle) became the most prevalent cycle on Earth (Fuchs, 2011; Liang et al., 2020).

## CARBOXYSOMES—THE DICE‐LIKE MARVELS FOR CONCENTRATING CO_2_

### Structural studies using cryo‐EM and cryo‐ET elucidate the architecture and mechanism of carboxysomes

In the past decade, unprecedented advances in structural biology have illuminated the architecture and assembly principles of carboxysomes. Using single‐particle cryo‐electron microscopy (cryo‐EM), cryo‐ET, and atomic force microscopy (AFM), near atomic‐scale details of shell protein matrices, Rubisco encapsulation, assembly intermediates, and permeability properties have been resolved (Evans et al., 2023; Huang et al., 2022; Kong et al., 2024; Sarkar et al., 2024; Sutter et al., 2019).

Carboxysomes are protein microcompartments built from thousands of proteins that form an icosahedral structure that is similar to cage shaped virus capsids (Kerfeld et al., 2005; Tanaka et al., 2008). High‐resolution cryo‐EM analysis has revealed that both α‐ and β‐carboxysomes possess shells composed of two main protein types: hexamer‐forming proteins (BMC domain‐containing, for example, α‐carboxysomes: CsoS1A‐C; β‐carboxysomes: CcmK2/CcmK4) and pentamer‐forming proteins (CsoS4/CcmL) (Ni et al., 2022; Sarkar et al., 2024). The hexamers tile the majority of the shell surface, forming 20 triangular facets that create semi‐permeable barriers, while the pentamers cap the 12 vertices of the icosahedron. The orientation of hexamer and pentamer, as well as their intermolecular contacts, define the shell's selectivity and mechanical stability (Sutter et al., 2019).

**Figure 1:**
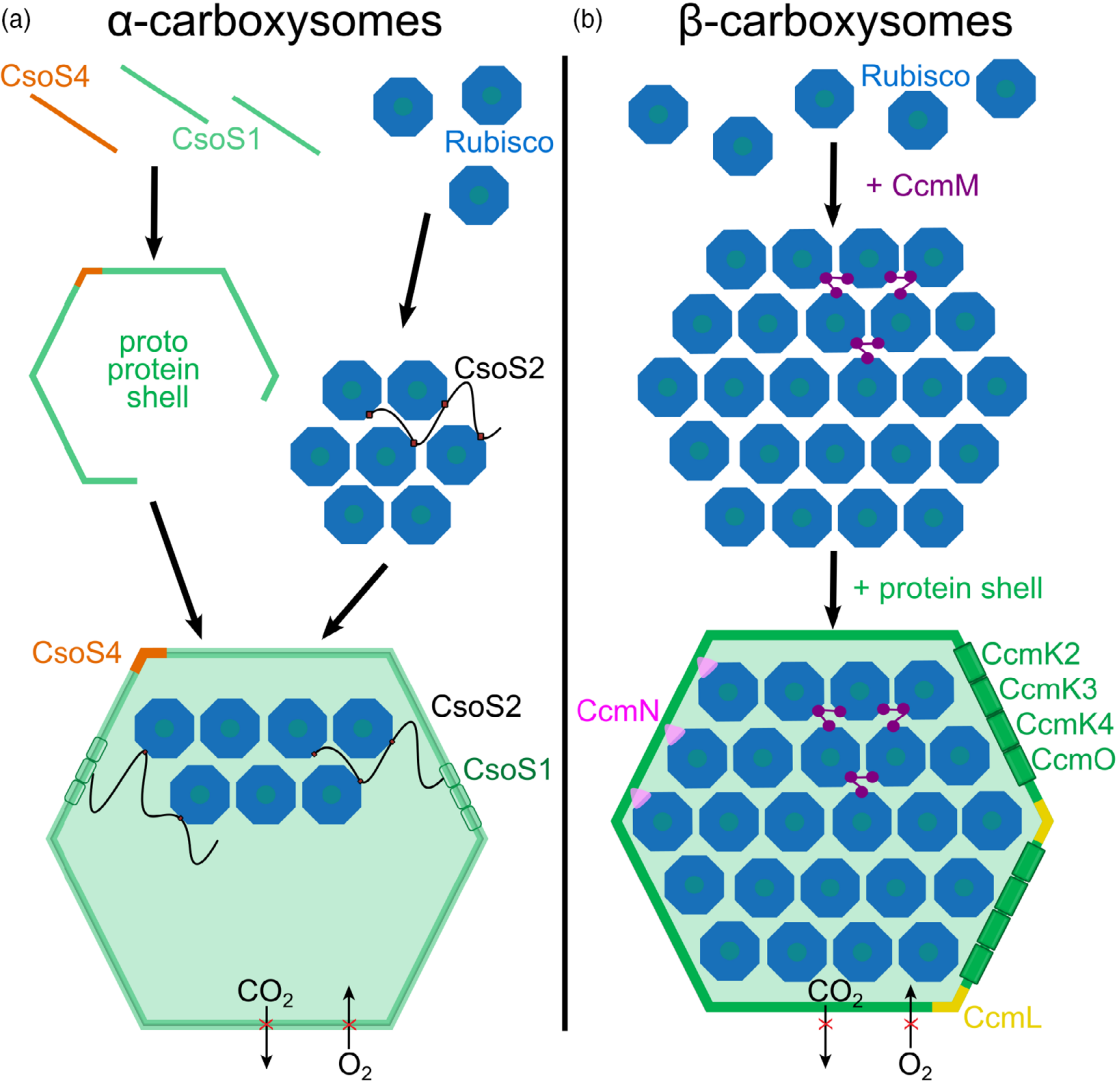
The assembly pathway of α‐ and β‐carboxysomes. (a) The shell and cargo of α‐carboxysomes can assemble concurrently. The Rubisco assembly formation is facilitated by the intrinsically disordered linker protein CsoS2, and the protein shell is composed of hexamer proteins CsoS1 and pentamer proteins CsoS4. CsoS4 also forms pores enabling molecular transit through the shell. (b) In contrast, β‐carboxysome assembly follows a ‘core first, shell second’ mechanism, beginning with Rubisco packing facilitated through the scaffolding protein CcmM. When the microcompartment reaches a critical size, CcmN mediates the interaction with the shell proteins CcmK2, CcmK3, CcmK4, CcmO (hexamers) and CcmL (pentamer).

In native α‐carboxysomes (Figure 1a), the hexamers form pores at their central axes, which facilitate controlled molecular transit, and are further joined by pseudohexameric proteins, such as CsoS1D, that are presumed to act as gated conduits for the transport of ribulose‐1,5‐bisphosphate (RuBP) and other larger metabolites via stacked trimers forming an airlock. Additionally, subtle charge differences in CsoS1A versus CsoS1C hexamers result in variable selectivity for negatively charged metabolites. Via shell protein mutations and engineered variants, this permeability has been experimentally modulated (Li et al., 2020; Sun et al., 2022; Wang et al., 2024; Huang et al., 2022; Faulkner et al., 2020).

Inside the α‐carboxysome, the intrinsically disordered protein (IDP) CsoS2 determines architecture and assembly dynamics. CsoS2 contains multiple tandem repeat domains in its N‐terminal region, each capable of binding to a conserved surface of the Rubisco holoenzyme by engaging specific acidic and hydrophobic residues on Rubisco's large and small subunits (Blikstad et al., 2023; Ni et al., 2022, 2023; Oltrogge et al., 2020; Turnšek et al., 2024). This positions Rubisco in a tight matrix or array formation within the lumen of the carboxysome. Structural and mutational studies have shown that around 10–13 CsoS2 repeats (four N‐terminal, six middle region, three C‐terminal in *Halothiobacillus neapolitanus*) per linker may be present, each acting as a modular tether (Cai et al., 2015; Ni et al., 2023; Oltrogge et al., 2020). Both repeat number and post‐translational modifications finely regulate the affinity of interaction. Simultaneously, the C‐terminal region of CsoS2 interacts with the inner facets of the shell proteins, specifically conserved grooves on CsoS1 (hexamers) and CsoS4 (pentamers), through conserved [I/V]TG motifs which dock precisely into the threefold junctions between CsoS1As and CsoS1A/CsoS4A interfaces. This docking is mediated by hydrogen bonds ([I/V]TG → CsoS1A H79, CsoS2 R855 → CsoS4A H21) and salt bridges (CsoS2 R731 → CsoS4A E22, CsoS2 R784 → CsoS4A E69), which effectively anchor the Rubisco condensate to discrete locations on the shell (Ni et al., 2023; Oltrogge et al., 2024).

The pairwise coordination between Rubisco subunits and the CsoS2 repeats forms local enzyme clusters, which yield a higher‐order concentric organization into several layers, as visualized by cryo‐ET (Ni et al., 2022). This layering generates structurally distinct zones within the carboxysome that may influence substrate channeling and metabolic flux. The spatial proximity enforced by CsoS2 is argued to accelerate the delivery of CO_2_ produced by the shell‐localized CA to Rubisco's active sites.

Biophysical experiments based on *in vitro* reconstitution systems with Rubisco and an N‐terminal fragment of CsoS2 have demonstrated that the resulting Rubisco‐CsoS2 condensate exhibits properties of a liquid‐like phase (Oltrogge et al., 2020), marked by rapid molecular exchange and dynamic rearrangement, which could be crucial for organelle repair, turnover, and adaptability in response to changing cellular CO_2_ levels as hypothesized for pyrenoids (Freeman Rosenzweig et al., 2017; Hill et al., 2020; Huffine et al., 2024; Niederhuber et al., 2017; Zang et al., 2021). Remarkably, this condensate can self‐assemble independently of the shell structure: purified CsoS2 and Rubisco spontaneously form dense condensates, while shell protein mixtures can assemble into hollow capsids under physiological conditions in the absence of cargo. These findings illustrate the inherent modularity and self‐sufficiency of the matrix and the shell (Figure 1a) (Menon et al., 2008; Oltrogge et al., 2024; Wang et al., 2024).

*In vivo* and synthetic systems have further revealed that fusion of additional M‐regions, or mutation of CsoS2 domains, can dramatically alter carboxysome size, cargo density, and permeability, providing evidence for a tunable assembly process driven by the scaffold's sequence and structure (Li et al., 2024; Long et al., 2018; Oltrogge et al., 2024).

In contrast to α‐carboxysomes, β‐carboxysome biogenesis proceeds via a step‐wise assembly (Figure 1b), in which Rubisco packing and microcompartment construction are directed by the scaffold protein CcmM. CcmM's two isoforms, M58 and M35, which are produced through alternative translation initiation, contain multiple small subunit‐like (SSUL) domains that are homologous to Rubisco's small subunit, as well as a C‐terminal γ‐CA domain in M58 (Ryan et al., 2019). These SSUL domains promote the nucleation and organization of Rubisco into tightly packed, paracrystalline arrays that exhibit pseudo‐hexagonal symmetry in the core of the carboxysomes (Sun, Sheng, et al., 2025). Each CcmM molecule can simultaneously bind several Rubiscos, aligning them in near‐hexagonally packed arrays that maximize Rubisco concentration (Kong et al., 2024).

After Rubisco assemblies reach a critical size, the encapsulation machinery is recruited. CcmN contains a unique linker peptide at its C‐terminus (~17–18 amino acids forming an amphipathic α‐helix) that mediates the interaction between the Rubisco‐CcmM matrix and the following shell proteins: CcmK2, CcmK3, CcmK4, CcmL, and CcmO (Kinney et al., 2012; Lin et al., 2014; Sun et al., 2021). The shell proteins self‐assemble around these pre‐formed cores facilitated by CcmN, resulting in the canonical icosahedral morphology but with variable facet composition (i.e., stoichiometry of paralogs) depending on organism and environmental signals (Figure 1b). While the α‐carboxysome shell and cargo assemble concurrently, β‐carboxysomes rely on this ‘core first, shell second’ mechanism, such that empty shells rarely form (Cameron et al., 2013; Sutter et al., 2019).

On a nanoscale, β‐carboxysomes can reach diameters of 100–400 nm, dwarfing their α‐carboxysome counterparts, which typically range from 40 (engineered) to 160 nm (Oltrogge et al., 2024). This larger size is attributed to the capacity for the formation of large Rubisco clusters through CcmM and the shell not being able to self‐assemble (Whitehead et al., 2014). Thus, β‐carboxysomes can contain ~1.5 times more Rubisco complexes compared to α‐carboxysomes (Whitehead et al., 2014; Sun et al., 2022; Faulkner et al., 2017; Ni et al., 2022; Niederhuber et al., 2017; Sun, Sheng, et al., 2025).

Carboxysomes, while unique in their CO_2_ concentrating function, share core architectural principles with other bacterial microcompartments (BMCs), such as propanediol utilization BMC, ethanolamine utilization BMC, or glycyl radical enzyme‐containing BMC. Comparative studies show conservation in BMC hexamer and pentamer motifs, permeability determinants, and shell formation strategies (Li et al., 2021; Ochoa et al., 2021; Raza et al., 2024; Sutter et al., 2021; Trettel et al., 2023; Young et al., 2025).

### Biochemical characterization of catalytic mechanism

The selectively permeable shell of carboxysomes creates a microenvironment that concentrates CO_2_ near Rubisco's active site while minimizing the competing oxygenation reaction that leads to photorespiration (Faulkner et al., 2020; Sarkar et al., 2024). The fundamental principle underlying carboxysome function involves the uptake of bicarbonate (HCO_3_^−^) from the cytoplasm through pores in the shell. This process utilizes the 50–1000‐fold approximate up‐concentration of bicarbonate in the cytosol compared to the environment and, thus, yields a saturated inner lumen of the carboxysomes (Flamholz et al., 2020; Huang et al., 2022).

Inside the carboxysome, CA catalyzes the rapid conversion of HCO_3_^−^ to CO_2_ and H_2_O, generating local CO_2_ concentrations that can be 10–100 times above ambient levels (Mangan & Brenner, 2014). This enables Rubisco to operate near its maximum carboxylation rate with minimized oxygenation activity (Flamholz et al., 2022; Long et al., 2018; Mangan & Brenner, 2014). The specific protein–protein interactions between disordered regions and Rubisco creates enzyme supercomplexes that presumably optimize substrate channeling and catalytic efficiency (Blikstad et al., 2023). In α‐carboxysomes of *Cyanobium* the CA CsoSCA, unlike the CA from the chemoautotrophic bacterium *H. neapolitanus*, displays additional allosteric activation by RuBP, which suggests a sophisticated regulatory mechanism that coordinates the activities of both enzymes (CA & Rubisco) within the carboxysome microenvironment (Pulsford et al., 2024).

On the edge of the carboxysome, the shell proteins create pores with selective permeability for charged metabolites such as HCO_3_^−^ and 3‐phosphoglycerate. Mathematical modeling and experimental studies have demonstrated that optimal carboxysome function requires fine‐tuned and rapid HCO_3_^−^ influx while minimizing CO_2_ leakage (Faulkner et al., 2020; Huang et al., 2022; Mahinthichaichan et al., 2018). Recent evidence, however, also suggests that CO_2_ retention rather than selective exclusion of oxygen may be the primary mechanism for CCM efficiency (Mangan et al., 2016). *In vivo* biochemical investigations of carboxysome function have shown that CA activity must be strictly compartmentalized within the carboxysome, as cytosolic expression of CA destroys CCM function by dissipating the bicarbonate gradient before it can be utilized for CO_2_ up‐concentration (Flamholz et al., 2020, 2022; Price & Badger, 1989b).

Beyond the shell, the apparent catalytic parameters of encapsulated Rubisco appear to differ significantly from those of the free enzyme. Experimental studies have demonstrated that carboxysome‐encapsulated Rubisco exhibits approximately twofold higher apparent Michaelis–Menten constants for RuBP compared to the free enzyme, with K_M_ values increasing from approximately 36 ± 1 μM for free Rubisco to 59 ± 3 μM for the carboxysome‐encapsulated Rubisco (Long et al., 2018). This indicates that the proteinaceous shell creates a diffusional barrier that restricts RuBP access to the enzyme's active site. This elevated K_M_ for RuBP has been observed in α‐carboxysomes isolated from native cyanobacteria, as well as those reconstituted in transgenic plants and is suggested to be consistent across both α‐ and β‐carboxysomes, suggesting a fundamental consequence of encapsulation rather than species‐specific effects (Long et al., 2018, 2021; Orr et al., 2020; Whitehead et al., 2014). Restricted substrate access appears to result from the selective permeability properties of the shell proteins, particularly the hexameric BMC domain‐containing proteins. These accommodate the passage of the bulkier RuBP molecule through channels, which also must restrict the efflux of CO_2_. Molecular dynamics simulations suggest that RuBP exhibits the slowest permeability coefficient among all CBB cycle intermediates tested, with permeability values approximately 10‐fold lower than smaller metabolites such as 3‐phosphoglycerate (Long et al., 2021; Rae et al., 2013). It appears that the three‐dimensional structure and charge distribution of RuBP creates significant energetic barriers for transit through the shell pores. In contrast to the restricted RuBP kinetics, the authors state that carboxysome‐encapsulated Rubisco displays CO_2_ kinetics that are remarkably similar to those of the free enzyme, with K_M_(CO_2_) values (Michaelis–Menten constants for CO_2_) remaining largely unchanged upon encapsulation (around 250 μM at standard atmosphere) (Long et al., 2018; Sarkar et al., 2024).

The authors further argue that the preservation of K_M_(CO_2_) suggests for the carboxysome shell not to significantly impede CO_2_ diffusion, consistent with molecular simulations showing that CO_2_ has one of the highest permeability coefficients across the shell (~10^−2^ cm s^−1^). This stands in contrast to its presumed role as a diffusional barrier for CO_2_ (Faulkner et al., 2020; Sarkar et al., 2024). Furthermore, the turnover number (*k*_cat_) of encapsulated Rubisco shows similar to slightly elevated catalytic rates despite the confinement, indicating that the high local enzyme concentration may partially compensate for reduced substrate accessibility. It is not clear if the CO_2_/O_2_ specificity factor (S_C/O_) of encapsulated Rubisco generally remains similar to that of the free enzyme (Long et al., 2018; Sarkar et al., 2024). In theory, the apparent specificity should differ from the free enzyme because the elevated CO_2_ concentrations achieved inside should effectively suppress oxygenation activity by mass action according to the current biochemical model. Computational modeling efforts suggest this theory by estimating a changed apparent S_C/O_, showing dramatically enhanced carboxylation efficiency under physiological conditions with over 2600 CO_2_ molecules fixed for every molecule that escapes the compartment (Sarkar et al., 2024).

Beyond the canonical mechanism of carboxysomes, they display a remarkable diversity as evidenced by the recently identified iota‐class CAs (iCA) in sulfur‐oxidizing bacteria. This CA does not require a metal cofactor in the reaction center but relies solely on the protein structure (Wieschollek et al., 2024; Yadav et al., 2024). Additionally, systems‐level analysis indicates that carboxysomes may function as part of a larger cellular metabolon. In this metabolon, physically associated enzymes facilitate metabolite channeling and enhance carbon flux through the CBB cycle, with the carboxysome serving as a central hub that coordinates multiple metabolic pathways involved in CO_2_ fixation (Abernathy et al., 2019; Huffine et al., 2023).

## PYRENOIDS—THE MICROBIAL EUKARYOTIC SOLUTION FOR CONCENTRATING CO_2_

### Macro structure resolved by cryo‐ET shows internal organization

**Figure 2:**
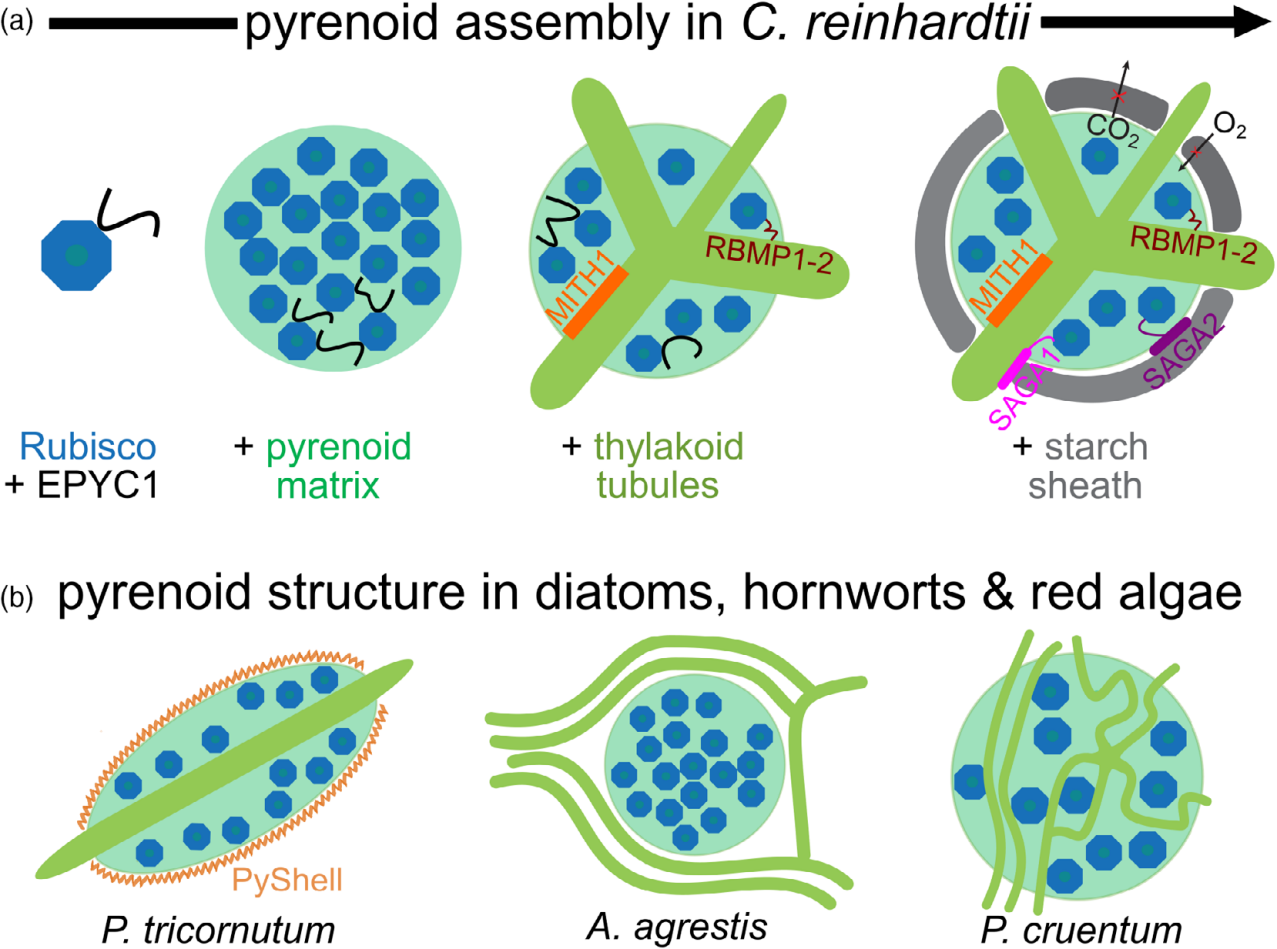
The architecture of pyrenoids in various species. (a) The architecture and assembly pathway of pyrenoids in *Chlamydomonas reinhardtii*. Rubisco is aggregated by the intrinsically disordered protein EPYC1, creating a so‐called pyrenoid matrix. In the next step, thylakoid tubules are drawn through the pyrenoid by MITH1, while RBMP1‐2 mediates an interaction between the matrix and the thylakoid tubules. Finally, the pyrenoid is enveloped by a starch sheath. This process is facilitated by SAGA1 and SAGA2, which bind the starch plates and promote interaction between the matrix and the shell. (b) Examples of different pyrenoid architectures occurring in the model diatom *Phaeodactylum tricornutum*, model hornwort *Anthoceros agrestis*, and red alga *Porphyridium cruentum*.

Although most algae and hornwort species possess pyrenoids, they are not a mandatory feature. They come in a diverse set of organizations ranging from one to tens of pyrenoids per chloroplast and appear to be more structurally flexible than carboxysomes (Neofotis et al., 2021; Robison et al., 2025; Villarreal & Renner, 2012). They consist either of the pyrenoid matrix alone—a highly dense structure of Rubiscos—or of the matrix traversed by thylakoid membranes and can additionally be enveloped by a starch sheath or protein shell (Atkinson et al., 2024; Engel et al., 2015; He et al., 2023; Hennacy et al., 2024; Itakura et al., 2019; Nam et al., 2024). In the model green alga *Chlamydomonas reinhardtii*, pyrenoid constitution and assembly have been studied extensively, which has shown that Rubisco is first condensed by the IDP EPYC1 creating the pyrenoid matrix, which is then transversed by thylakoid tubules and finally enveloped by a starch sheath (Figure 2a). However, it has been observed that the pyrenoid architecture can vary significantly across species. For example, the model diatom *Phaeodactylum tricornutum* consists of a pyrenoid matrix that is transversed by only a single thylakoid tubule and is surrounded by a proteinaceous sheath of pyrenoid shell (PyShell) proteins, while hornworts such as *Anthoceros agrestis* are not transversed by thylakoid tubules but instead are surrounded by them. The red alga *Porphyridium cruentum* on the other hand exhibits a pyrenoid matrix transversed by a highly complex network of interconnected tubules without a surrounding sheath (Figure 2b) (Barrett et al., 2021; McKay & Gibbs, 1990; Robison et al., 2025; Shimakawa et al., 2024).

Inside the pyrenoid, Rubisco is tightly packed into hexagonal or cubic formations elucidated by cryo‐ET studies of the *C. reinhardtii* pyrenoid (Engel et al., 2015). Using modified *in situ* cryo‐ET and fluorescence recovery after photobleaching for ultrastructure elucidation of the pyrenoid, the formation of a liquid‐like droplet was shown, marked by rapid molecular exchange and dynamic rearrangement (Freeman Rosenzweig et al., 2017). The mobility of the main protein constituents of the *C. reinhardtii* pyrenoid—Rubisco and the IDP EPYC1—further underpins the liquidity. This rapid exchange appears to be necessary for organelle repair, turnover, and response to changing cellular CO_2_ levels (Freeman Rosenzweig et al., 2017). The prevalence of EPYC1 has also been demonstrated with proteomics, estimating that it is one of the most abundant proteins in the pyrenoid, playing a key role in the pyrenoid assembly (Mackinder et al., 2016, 2017; Wunder et al., 2018).

Unlike carboxysomes, in which a CA is located in the matrix, so that HCO_3_^−^ is converted to CO_2_ proximal to Rubisco, current evidence does not suggest a CA in the pyrenoid matrix (Fei et al., 2022). Instead, CAs are located in the lumen of thylakoid tubules that often spike into the pyrenoid matrix, or envelop it. These spiking or enveloping thylakoids show a remarkable range of diversity. The thylakoid sheets can surround the pyrenoid (i.e., in *A. agrestis* and some dinoflagellates), thylakoids can traverse the pyrenoid by forming a single disc that bisects the pyrenoid matrix (i.e., in *P. tricornutum* and several *Chlorella* species), or multiple non‐connecting, parallel stacks of thylakoids can pass through the pyrenoid (common in green algae and *Euglena*; Figure 2) (Kusel‐Fetzmann & Weidinger, 2008; Meyer et al., 2017; Treves et al., 2016). In *C. reinhardtii* the biogenesis of the spiking thylakoids is facilitated by SAGA1 on the matrix surface, followed by MITH1 drawing the membranes through the pyrenoid (Franklin et al., 2026; Hennacy et al., 2024). A suspected advantage of the segregation of Rubisco and the CA is that both enzymes can operate at their respective optimal pH levels (Fei et al., 2022; Robison et al., 2025).

Surrounding the pyrenoid, presumably to prevent CO_2_ leakage, *C. reinhardtii* and many other algae lineages develop a starch sheath, which has been resolved through cryo‐ET (Engel et al., 2015; Fei et al., 2025). Nonetheless, some pyrenoid‐containing organisms do not feature such an envelope. Instead of starch sheaths, diatoms appear to use a protein shell (Nam et al., 2024; Shimakawa et al., 2024), while hornwort pyrenoids are surrounded by thylakoid stacks (Robison et al., 2025). It is not fully understood, if the starch sheath truly prevents the CO_2_ leakage as is also disputed for the carboxysome shells, or if the enveloping structures have more architectural function as is suggested by the *saga1* mutant (Fei et al., 2025; Itakura et al., 2019).

Reverse genetic studies have further shown that not only a single pyrenoid but also many within one cell can be functional. For example, recent experiments have shown that *C. reinhardtii* cells that carry a mutation in the *SAGA1* gene have 10 pyrenoids on average. *C. reinhardtii* with the mutated *SAGA1* gene remains viable; however, as the mutation leads to a reduced photosynthetic efficiency, the mutant is only cultivatable in high CO_2_ concentrations (Itakura et al., 2019). A naturally occurring example is the species *Scytosiphon lomentaria*. In this species, a second pyrenoid is formed *de novo* in the parental chloroplast prior to cell division, and subsequently the two pyrenoids are passed equally to the daughter cells upon division (Nagasato & Motomura, 2002). While most algae have only one pyrenoid, hornworts in general (i.e., *A. agrestis*) have multiple pyrenoids per chloroplast displaying considerable variation with ~25 pyrenoids observed in *Phaeoceros laevis* (Griffiths, 1970; Robison et al., 2025; Villarreal & Renner, 2012).

While the pyrenoid matrix is mainly constituted from Rubisco and EPYC1, and Rubisco specifically being the main protein observable with cryo‐ET, proteomics studies of *C. reinhardtii* revealed that a total of 190 proteins are within the pyrenoid proteome (Zhan et al., 2018). Among the most abundant proteins in the *C. reinhardtii* proteome is Rubisco activase, which is often necessary for the continued activity of form I Rubiscos. This observation shows that similar to carboxysomes, in which Rubisco activases are at least suspected, the pyrenoid needs to sequester significant repair machineries to keep the organelle running (Sun et al., 2022). In contrast to green algae, the activase activity in non‐green algae is taken on by the chaperone‐like Rubisco activase CbbX (Mueller‐Cajar et al., 2011; Kroth, 2015).

Even though *C. reinhardtii* has been studied most extensively, investigating other species has shown that the proteins involved in pyrenoid assembly may differ significantly from one species to another. While functional homologs to EPYC1 are also present in the genomes of other algae with pyrenoids, such as PYCO1 in diatoms (Oh et al., 2023) and CsLinker in chlorella (Barrett et al., 2024), they do not share any sequence homology. A recent study into the pyrenoid proteome of the chlorarachniophyte alga *Amorphochlora amoebiformis* identified the matrix protein PPAP28 as a potential Rubisco‐binding linker protein. Since its homologs have not been detected in other chlorarachniophytes, the study suggested that PPAP28 is even species‐specific (Moromizato et al., 2024). Such findings underline that the macro‐structures of pyrenoids are highly diverse despite serving the same purpose and that even down to the individual protein homology can be minimal.

### Reverse and forward genetics reveal the function of individual components

Using reverse and forward genetics, the functional components of pyrenoids in *C. reinhardtii*—the matrix, the shell and the tubules—have further been elucidated in recent years. These three essential CCM modules for the pyrenoid have been proposed based on phenotypic characterizations coupled to genetic deletions (for example the requirement for more ATP per CO_2_ molecule fixed and/or a diminished capacity to concentrate inorganic carbon) (Adler et al., 2022; Dao et al., 2025; Fei et al., 2022).

The first module represents a chloroplast CO_2_ uptake system that uses either a stromal CA to facilitate the diffusive influx of CO_2_ and conversion into HCO_3_^−^ (i.e., the LCIB‐dependent pathway) or an active pump to import HCO_3_^−^ (i.e., HLA3/LCIA‐mediated HCO_3_^−^ uptake). The LCIB‐dependent pathway is active under low CO_2_ conditions, during which LCIB is dispersed throughout the chloroplast stroma. The mechanistic importance under low CO_2_ conditions has been elucidated through *lcib* mutants (Δ*ad1*), which exhibit impaired photosynthesis and C_i_ transport (Duanmu et al., 2009; Wang & Spalding, 2014; Yamano et al., 2015).

The second module involves a system that transports HCO_3_^−^ into the thylakoid lumen through channels in the thylakoid membrane, enabling HCO_3_^−^ to diffuse into the tubules within the pyrenoid matrix, where it is converted back into CO_2_ by a CA. The main channels for HCO_3_^−^ transport from the chloroplast stroma to the thylakoid lumen are considered to be bestrophin‐like proteins (BSTs), which are located on the thylakoid membrane. RNAi mutants with reduced expression of all three BST gene paralogous (BST1‐3) were unable to survive under low CO_2_ conditions (Mukherjee et al., 2019). BST4 has been proposed to be an additional HCO_3_^−^ channel, with a particular importance during the transition from dark to light and for the regulation of the luminal pH in *C. reinhardtii* (Adler et al., 2023). Additionally, the importance of a CA (CAH3) has been demonstrated by the *Cah3* mutant, which exhibits impaired CCM function (Sinetova et al., 2012). The thylakoids transversing the pyrenoids are facilitated by SAGA1 on the matrix surface, followed by MITH1 drawing the membranes through the pyrenoid. Furthermore, Rubisco‐Binding Membrane Proteins 1‐2 (RBMP1‐2) have been found to be essential by mediating interactions between the dense matrix and the thylakoid tubules (Adler et al., 2023; Meyer, Itakura, et al., 2020).

The third module represents a dense Rubisco core, that is, the pyrenoid matrix. This matrix is in the most studied pyrenoids formed through co‐condensation of Rubisco with an associated liquid–liquid phase separation (LLPS) prone IDP. In *C. reinhardtii*, the essential function of EPYC1 in forming the pyrenoid matrix has been observed using fluorescence microscopy combined with reverse genetics, as *epyc1* mutants showed a misallocation of Rubisco, with a large fraction found outside the pyrenoid. This results in decreased CO_2_ fixation through Rubisco, which explains the severe CCM defect observed in this mutant (He et al., 2020; Mackinder et al., 2016).

Pyrenoids are often surrounded by a defined physical barrier, which can be composed of starch plates (*C. reinhardtii*), a protein shell (diatoms) or thylakoid stacks. It has been hypothesized that this structure acts as a diffusional barrier to CO_2_ and O_2_. This effect has been supported by the *C. reinhardtii* mutant *sta2‐1* that develops thinner starch plates due to a defective granule‐bound starch synthase I and exhibits lower CCM efficiency at concentrations of CO_2_ below ambient air (Toyokawa et al., 2020). Furthermore, the starch sheath appears to be involved in pyrenoid assembly, providing structural support (Atkinson et al., 2024; Barrett et al., 2021; Itakura et al., 2019). Several proteins have been identified as playing a role in the formation and maintenance of the starch sheath. Among these are SAGA1 and SAGA2 (Starch Granule Abnormal 1 and 2), which have been shown to bind to the starch plates and mediate interaction between the matrix and shell via a Rubisco‐binding motif (He et al., 2023; Meyer, Itakura, et al., 2020). The variety of proteins observed to be involved in pyrenoid assembly, as determined through physiological and genetic experiments, supports the observation that pyrenoids evolved independently multiple times (Long et al., 2024; Moromizato et al., 2024).

In contrast to the reverse genetic experiments using knock‐outs/knock‐downs, transplantation efforts of the pyrenoid into plants have shown the extent as well as the limits of our understanding of pyrenoid function. While a considerable proportion of the CCM components of *C. reinhardtii* appear to be compatible with plants, at least regarding their appropriate localization (Atkinson et al., 2016), a fully functional pyrenoid has not yet been achieved in plant chloroplasts (Catherall et al., 2025). The first successful step was the expression of a proto‐pyrenoid in the chloroplasts of *Arabidopsis thaliana*. This proto‐pyrenoid consisted of EYPC1 and a plant–algal hybrid Rubisco (combining *A. thaliana* RbcL and *C. reinhardtii* RbcS). Using this engineered system, pyrenoid‐like Rubisco condensation was observed (Atkinson et al., 2020). SAGA1 and SAGA2, responsible for recruiting starch around the pyrenoid matrix were successfully expressed in transgenic *A. thaliana* proto‐pyrenoid lines which led to large, plate‐like starch granules being observed around the edges of the matrix (Atkinson et al., 2024). The coexpression of MITH1 (Missing THylakoids 1) and SAGA1 in transgenic *A. thaliana* proto‐pyrenoid lines successfully led to the formation of thylakoid membranes traversing through the proto‐pyrenoid matrix (Catherall et al., 2025). However, their appearance was similar to plant thylakoid membranes, which suggests that there are still components involved in tubule formation and maturation that need to be identified.

### *In vitro* reconstitution underpinning catalytic mechanism

Using enzymatic assays to assess the CO_2_ fixation rates already show the effectiveness of minimal component pyrenoids. While recent studies have mostly focused on the *in vivo* formation and function of pyrenoids in *C. reinhardtii*, as well as their transplantation into plants, other studies have examined the efficiency of *in vitro*‐built minimal component pyrenoids, using a bottom‐up approach (Küffner et al., 2024; Wunder et al., 2018). These minimal pyrenoids consist of a simple bacterial Rubisco, namely form II Rubisco from *Rhodospirillum rubrum*, and a *C. reinhardtii* EPYC1. Interestingly, only EPYC1 induced the formation of condensates compared to other LLPS‐associated IDPs, emphasizing its role in pyrenoid assembly. Furthermore, Rubisco activity assays showed that the carboxylation rate of the Rubisco enzyme was significantly higher within the condensates compared to its rate when it was not phase separated. These results suggest that pyrenoids may have evolved from simple phase‐separating Rubisco/EPYC1 complexes, which raises questions about the more complex assemblies and the evolution of modern pyrenoids.

The minimal bottom‐up approach provides a blueprint for developing synthetic CO_2_‐concentrating organelles in plants using IDP‐mediated phase separation principles. In addition, this approach can be employed to further develop pyrenoids consisting of EPYC1 and form I Rubiscos, such as RbcLS from *C. reinhardtii*.

## HCO_3_ ^−^ AND CO_2_ TRANSPORTERS—THE ESSENTIAL WORKERS OF CCMs

While much focus has been directed on the CO_2_ concentrating organelles themselves, both carboxysome‐based CCMs and pyrenoid‐based CCMs need specialized carbonate and bicarbonate transporters for the elevation of the intracellular C_i_ concentration prior to CO_2_ fixation (Findinier & Grossman, 2023; Price et al., 2011; Rottet et al., 2024). The structural and functional diversity among these transporters reflects adaptations to different cellular architectures and environmental niches, but all converge on the same core principles: active accumulation of HCO_3_^−^, strategic placement relative to the CO_2_‐fixing microcompartment, and carbonate transport regulation mechanisms to optimize photosynthetic efficiency.

**Figure 3:**
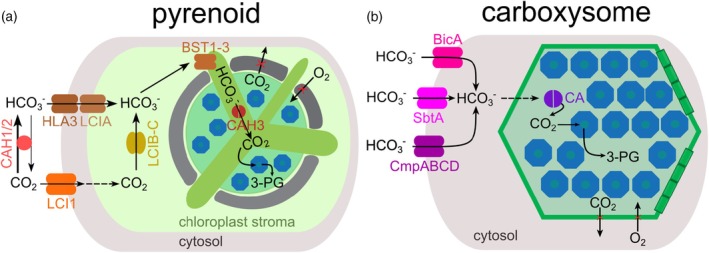
Different carbonate transporters in pyrenoids and carboxysomes. (a) In pyrenoids, HLA3 and LCIA actively pump HCO_3_^−^ across the plasma membrane, which is the main route for carbon uptake. The lesser‐used LCI1 channels provide passive CO_*2*_ influx, which is then converted into HCO_3_^−^ by LCIB and LCIC. The HCO_3_^−^ is then transported across the thylakoid membrane via the channels BST1‐3 and then rapidly converted to CO_2_ by the carbonic anhydrase (CA) CAH3. (b) In carboxysomes, HCO_3_^−^ is transported across the plasma membrane by the two sodium‐dependent transporters BicA and SbtA and the ATP‐binding cassette transporter CmpABCD. HCO_3_^−^ then enters the carboxysomes via pores and is converted by the CA CsoSCA (α‐carboxysomes) or γ‐CA domain/β‐CA CcaA (β‐carboxysomes).

In cyanobacteria, carboxysome‐dependent CCMs rely on a suite of C_i_ uptake systems localized to the plasma membrane and sometimes the thylakoid membranes. The major transporters include BicA (the low‐affinity, high‐flux sodium‐dependent bicarbonate transporter), the high‐affinity SbtA (sodium‐dependent), and CmpABCD (an ATP‐binding cassette transporter for bicarbonate) (Figure 3a) (Omata et al., 1999; Price et al., 2004; Price & Howitt, 2014). SbtA from *Synechocystis* functions as a trimeric elevator‐type transporter, shuttling HCO_3_^−^ and Na^+^ across the membrane via coordinated substrate‐binding and large conformational changes. SbtA forms a stable complex with SbtB, which regulates SbtA's activity allosterically through AMP or cAMP binding (Fang et al., 2021; Selim et al., 2018; Shibata et al., 2002). The T‐loop of SbtB locks SbtA in the inward‐facing conformation (Liu et al., 2021). BicA, on the other hand, operates as a dimer with a C‐terminal STAS domain required for activity and interacts with unknown regulatory proteins. These interactions may enable dynamic responses to changes in external and internal C_i_ concentration (Chan et al., 2022; Kurkela, 2020; Wang et al., 2019). The CmpABCD transporter in cyanobacteria consists of four subunits with distinct domains: CmpA is a periplasmic substrate‐binding lipoprotein that uses a unique pocket and a coordinating calcium ion for specific high‐affinity bicarbonate binding. CmpB forms the membrane channel, while CmpC and CmpD are ATP‐binding domains that power the transport (Badger & Price, 2003). This configuration allows selective, high‐affinity transport of HCO_3_^−^, tightly modulated by substrate availability and cytoplasmic ion balance (Koropatkin et al., 2007; Price et al., 2011).

In contrast to HCO_3_^−^ transport, uptake of CO_2_ does not play a major role for the cyanobacterial CCM. While CO_2_ is imported under very low external CO_2_ conditions, through CO_2_ uptake systems (NDH‐13/NDH‐14 complexes), it is not a principal driver of the CCM (Price et al., 2013).

In eukaryotic algae, pyrenoid‐based CCMs have evolved with a distinctly different set of transporters in accordance with subcellular compartmentalization. In *C. reinhardtii* and many diatoms, CO_2_ and bicarbonate transport is coordinated across the plasma membrane, chloroplast envelope, and thylakoid membranes (Figure 3b). The key plasma membrane CO_2_ channel (LCI1) provides passive CO_2_ influx, while active HCO_3_^−^ pumps first transport bicarbonate into the cytosol (HLA3 in *Chlamydomonas*, SLC4 in diatoms), and then into the stroma (LCIA in *Chlamydomonas*, unidentified in diatoms) (Kono et al., 2020; Nakajima et al., 2013; Yamano et al., 2015). Once inside the stroma, bestrophin‐like channels (BST1‐3) facilitate HCO_3_^−^ movement into the thylakoid lumen, where the luminal CA CAH3 rapidly converts it to CO_2_ (Mukherjee et al., 2019). Thylakoid tubules traverse the pyrenoid matrix, acting as a pipeline for newly generated CO_2_ to reach Rubisco (Meyer et al., 2017).

Structurally, LCI1 and LCIA are integral membrane proteins with channel‐like topology, mediating passive CO_2_ flux under concentration gradients, but actively regulated by cellular C_i_ concentrations and environmental signals (Wang & Spalding, 2014). HLA3 and related pumps are members of the ABC (as Cmp in cyanobacteria) or SLC transporter families. Their ATPase domains couple ATP hydrolysis to substrate translocation and are vital for CCM function at low CO_2_ concentrations (Duanmu et al., 2009; Prieß et al., 2018). BST channels are structurally related to bestrophin Ca^2+^ channels, with pore architectures adapted for HCO_3_^−^ selectivity, and may form multimeric complexes to optimize ion flux (Bharill et al., 2014; Mackinder et al., 2017; Qu & Hartzell, 2008).

While both carboxysome‐based and pyrenoid‐based CCMs use an array of carbonate and bicarbonate transporters, carboxysome systems rely on bacterial‐type transporters and direct cytosolic accumulation, whereas the eukaryotic pyrenoid systems utilize a more diverse set of transporters that are distributed across multiple membranes and employ specialized thylakoids that funnel CO_2_ to Rubisco. Both systems rely on regulated permeability, spatial compartmentalization of CA activity, and dynamic transporter control in response to photosynthetic demand. Current and future engineering efforts to transplant CCMs into crops or industrial microorganisms are focusing on grafting these transporter modules alongside the microcompartment to reconstruct the full CCM for enhanced productivity.

## DISCUSSION—COMPARING DIFFERENT BIOLOGICAL SOLUTIONS TO THE CO_2_ CONCENTRATION LIMITATION

The striking structural divergence between carboxysomes and pyrenoids reflects convergent evolution under distinct evolutionary and ecological pressures. Both systems enable CO_2_ fixation in low‐CO_2_ aquatic habitats, yet arose independently (He et al., 2023; Long et al., 2024; Rae et al., 2013). It is speculated that the first oxygenic photosynthesis machinery in cyanobacteria or their ancestors may have emerged around ~2300 mya (Raven et al., 2017). However, the first functional carboxysomes are hypothesized to have evolved ~350 mya in prokaryotic cyanobacteria, along with the appearance of pyrenoids in algae, while the first pyrenoids in hornworts are thought to have evolved ~100 mya ago (Badger & Price, 2003; Long et al., 2024; Rae et al., 2013; Villarreal & Renner, 2012). Recent phylogenetic, structural, and proteomic studies suggest that both pyrenoids and the two major carboxysome types have evolved convergently and likely originated multiple times in different lineages (Long et al., 2024; Moromizato et al., 2024; Rae et al., 2013).

A central factor for driving this diversity appears to be the environmental niche of these organisms. Except for a single lineage of land plants (hornworts), carboxysome or pyrenoid‐containing organisms are aquatic photosynthetic organisms, meaning they are affected by the specific properties of CO_2_ in water. In aquatic environments, CO_2_ has a relatively low solubility and at typical seawater pH most dissolved inorganic carbon is present as HCO_3_^−^ rather than free CO_2_. Additionally, nutrient concentrations can fluctuate dramatically due to temperature, pH, and photosynthetic activity (Griffiths et al., 2017; Maberly & Gontero, 2017). Here, compartmentalizing the CO_2_‐fixation process inside protein microcompartments or LLPS organelles can elevate local CO_2_ concentrations around Rubisco. These solutions seem tailored to water's diffusional limits, buffering external fluctuations, and minimizing photorespiratory waste (Griffiths et al., 2017; Long et al., 2021; Maberly & Gontero, 2017; Sarkar et al., 2024).

In terrestrial habitats, vascular plants face fundamentally different constraints. Atmospheric gas exchange is rapid, and diffusion is orders of magnitude faster than in water (Boston et al., 1989; Osmond et al., 1981). These observations could explain why vascular plants evolved biochemical CCMs, such as the C4 and the CAM pathway, which differ substantially from biophysical solutions. The biochemical strategies spatially (C4) or temporally (CAM) segregate initial CO_2_ capture from Rubisco by exploiting energetically expensive reactions to create local maxima in CO_2_ concentration in specialized tissues or cellular compartments, notably bundle sheath cells in C4 plants (Dodd et al., 2002; Lopes et al., 2011). Currently, there is evidence for combining biophysical and biochemical CCMs for only a few algae species (i.e., *Ulva prolifera*) and the fresh water plant *Ottelia alismoides*, indicating that the two CCMs can be coordinated in a complementary manner depending on changing environmental conditions (Jiang et al., 2026; Zhang et al., 2025). Nonetheless, this appears to be a rare feature, and biophysical CCMs are thought to be the dominant pathway in marine algae (Matsuda et al., 2017). Similarly, there are aquatic and semi‐aquatic embryophytes, such as *Hydrilla verticillata* and *Pilularia americana* that employ both C3 and C4 fixation pathways (Keeley, 1999; Reiskind et al., 1997; Xu et al., 2012). In general, however, rather than focusing on microcompartmental partitioning, terrestrial land plants exploit the capacity for active transport, membrane barriers, and cellular specialization driven by tissue differentiation (i.e., bundle sheath cells). These approaches are assumed to be more adapted to arid land environments where water loss and desiccation risk are dominant and where leaf and plant structures must maximize net carbon gain while minimizing photorespiration and water loss (Bräutigam et al., 2017; Osborne & Sack, 2012; Zhou et al., 2018).

Evolutionary lineage constraints may also play a role because microbial CCMs are assumed to have evolved a long time after the first endosymbiotic event and after the evolution of the first land plants (with the exception of *Paulinella*) (Gabr et al., 2020; Griffiths et al., 2017). While Raven et al. (2017) propose that *Gloeobacter*, the basal extant cyanobacterium, may have had CCMs already at the time of the Great Oxygenation Event (2300 mya), based on phylogenomic studies incorporating both protein and nucleotide data, as well as relaxed Bayesian molecular clock approaches (Blank & Sánchez‐Baracaldo, 2010; Dillon & Castenholz, 1999; Olsson‐Francis et al., 2010; Sánchez‐Baracaldo, 2015; Sánchez‐Baracaldo et al., 2014; Schirrmeister et al., 2015, 2016), other research shows that cyanobacteria evolved after the cyanobacteria crown group at around 350 mya, with no basal or stem group cyanobacteria extant (Butterfield, 2015; Lyons et al., 2014). Eukaryotic biophysical CCMs on the other hand, are consensually considered to have first appeared across algal lineages approximately 350 mya and in hornworts around 100 mya (Badger & Price, 2003; Meyer et al., 2017; Villarreal & Renner, 2012). These estimates correspond to a period during which various photosynthetic plant lineages had already been established and, hence, far after endosymbiosis. Additionally, the evolutionary data indicates that pyrenoids evolved multiple times independently via convergent evolution and, in the case of hornworts, they were lost and regained at least five times (Meyer, Goudet, & Griffiths, 2020; Rae et al., 2013; Raven et al., 2017; Villarreal & Renner, 2012). In comparison, complex multicellular land plants are limited in their evolutionary potential by slower rates of organelle innovation, developmental constraints, and the high energetic burden of maintaining microcompartments at cellular scales appropriate for larger organisms. These considerations may also explain the absence of microbial‐like CCMs in almost all terrestrial plants (He et al., 2023; Rae et al., 2013; Villarreal & Renner, 2012).

Overall, we still lack an in depth and mechanistic understanding for the plethora of approaches to concentrate CO_2_. To overcome this lack of knowledge and to enhance the photosynthetic efficiency of land plants, there has been extensive research in the past years to integrate microbial CCMs into crop species for improved growth rate and yield. Morphologically correct α‐carboxysomes containing cyanobacterial Rubisco, as well as key linker and shell proteins, have been shown to support autotrophic growth and enhanced photosynthesis in tobacco at elevated CO_2_. Although the transgenic plants were unable to grow under ambient air, the study provides a proof‐of‐concept that functional CCMs can operate within a C3 chloroplast background (Chen et al., 2023). In addition to transplanting native cyanobacterial components of the CCM into plants, future studies could also implement directed evolution approaches and novel synthetic systems to create a plant‐based CCM (Nguyen et al., 2024). Complementary work on pyrenoid‐based CCMs investigates the minimal modules needed to increase the concentration of CO_2_ near Rubisco, emphasizing microcompartments and CA relocalization as the next milestones toward fully functional microbial CCMs in major crop species (Adler et al., 2022).

## OUTLOOK

Despite major progress enabled by high‐resolution structural biology, advanced microscopy, and modern molecular biology, crucial aspects of microbial CCMs remain only partially understood. Recent studies have resolved the atomic architecture of carboxysome and pyrenoid shells, mapped the spatial organization of Rubisco and CA, and reconstructed partially functional CCM modules in non‐native organisms. However, the mechanism of selective permeability remains opaque: it is not clear how carboxysome shell pores distinguish between anionic bicarbonate, uncharged CO_2_, O_2_, and at the same time larger metabolites such as RuBP. Quantifying the dynamic selectivity, gating behavior, and modulation of specific shell protein variants is necessary to fully understand carboxysome function and regulation.

Beyond selectivity, the evolutionary drivers underlying the diversity of CCMs pose deep biological questions. Why has nature repeatedly solved the Rubisco problem with such a variety of approaches? Environmental constraints, lineage‐specific genetic toolkits, and physical limitations seem to force the trajectories, but the exact causal factors and why higher plants never adopted microbial solutions or lack a CCM, such as C3 plants, remain unanswered.

A challenge for translation is engineering CCMs into non‐native, high‐yield phototrophic systems for agriculture and biotechnology. Recent synthetic biology approaches demonstrate architectural feasibility. However, attempts at creating functional heterologous reconstitutions in plants showing enhanced photosynthesis have mostly failed. In one case, reconstitution in tobacco succeeded but led to reduced growth in ambient air. This shows that building functional carboxysomes or pyrenoid‐like organelles in plants or microbes requires precise control over enzyme stoichiometry, shell assembly, metabolite transport, and cofactor regulation, highlighting our limited understanding of biophysical CCMs and, hence, poses major difficulties for applications. In conclusion, not only are further efforts into understanding the fundamental biochemistry and biophysics of CCMs needed, but also more work on the bottom‐up engineering of CCMs is required (Box 1).

Box 1 **Key remaining questions**

- How is the dynamic selectivity of biophysical CCMs mechanistically enforced?
- What are the mechanistic factors for the evolution of specific CCM architectures?
- How are the protein stoichiometry, CCM assembly, and regulation in native organisms controlled and how can this be applied to eventual transplantation into plants?

## CONFLICT OF INTEREST

None of the authors have a conflict of interest to disclose.

## Data Availability

Data sharing not applicable to this article as no datasets were generated or analysed during the current study.
